# Minilaparotomy Hysterectomy as a Suitable Choice of Hysterectomy for Large Myoma Uteri: Literature Review

**DOI:** 10.1155/2016/6945061

**Published:** 2016-01-27

**Authors:** Kenichiro Sato, Yasuyoshi Fukushima

**Affiliations:** Department of Obstetrics and Gynecology, Kyoaikai Hospital, 7-21 Nakajima-cho, Hakodate-shi, Hokkaido 040-8577, Japan

## Abstract

The objective of this paper is to propose minilaparotomy hysterectomy as the suitable choice for large uterus on the basis of our experienced case of performed minilaparotomy hysterectomy to 4,500 g myoma uteri and review published cases about this clinical condition. We presented a 44-year-old woman (gravida 0, virgin) who consulted our hospital because of the chief complaints of abnormal genital bleeding and hypermenorrhea. Transabdominal ultrasonography revealed that abdominal solid tumor reached over the navel. Her tumor was an indication of surgery; to do minilaparotomy hysterectomy with laparoscope was decided because her informed consent was obtained. A 6 cm transverse incision (Maylard incision) was made to the skin above the pubic hairline. At the end of surgery, the length of abdominal wound was 8.5 cm, operating time was 128 min, weight of resected myoma uteri was 4,500 g, and intraoperative blood loss was 895 mL. Blood transfusion was not done; postsurgical course was not a problem without anemia. We propose that a large uterine case in which it is difficult to perform vaginal or laparoscopic hysterectomy should be considered in order to select minilaparotomy hysterectomy up to around 5 kg weight of uterus, and the length of skin incision in minilaparotomy hysterectomy is necessarily <9 cm particularly in large uterus.

## 1. Introduction

Currently, primary methods of hysterectomy are vaginal, abdominal, laparoscopic, and robotic hysterectomy. In March 2013, the president of Obstetrics and Gynecology Society of United States made the statement that robotic surgery takes a high cost in hysterectomy of benign disease except complex surgery such as cancer, but the improvement of patient outcomes is not observed, so vaginal hysterectomy is the first choice and the second choice is laparoscopic hysterectomy [1]. However, vaginal hysterectomy or laparoscopic hysterectomy is not possible in all cases. Some cases have necessary to choose conventional abdominal hysterectomy. Recently, we experienced a 4,500 g myoma case that had done minilaparotomy hysterectomy; it is considered one of the minimally invasive surgeries. To our knowledge, at the present time this case is heaviest myoma that had done minilaparotomy hysterectomy, so this case will be helpful in understanding the limitations and indication of minilaparotomy hysterectomy. We will discuss suitable choice of hysterectomy with reference of this case and review of other case reports published about this clinical condition on the basis of uterine weight mainly.

## 2. Case Presentation

**Figure 1:**
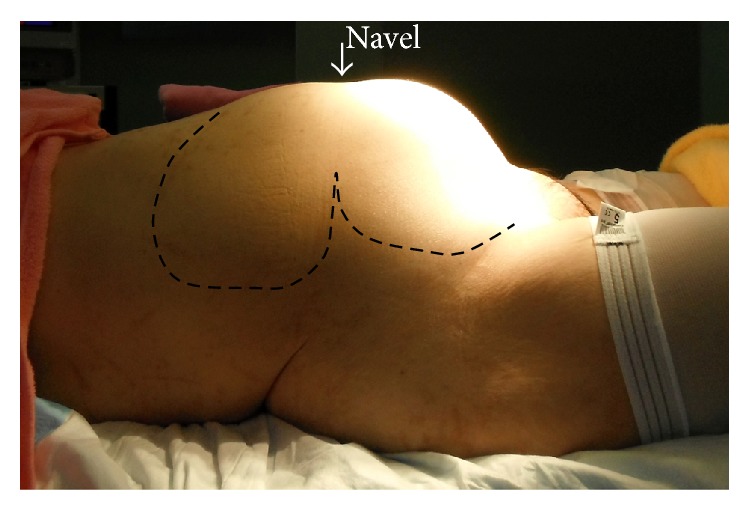
Abdominal tumor extending beyond the navel.

**Figure 2:**
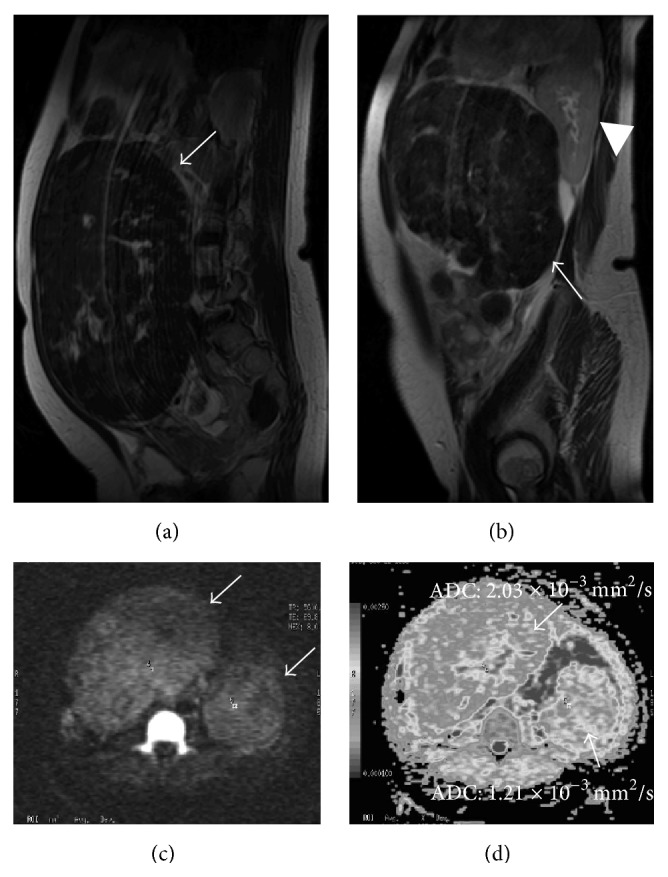
Two large tumors ((a–d), arrow) reached kidney ((b), arrow head). These tumors showed low intensity on T2-weighted image ((a) and (b), arrow) and intermediate intensity on diffusion-weighted image ((c), arrow). The apparent diffusion value was 2.03, 1.21 × 10^−3^ mm^2^/s ((d), arrow); thus, we did not consider that these tumors were as leiomyosarcoma.

**Figure 3:**
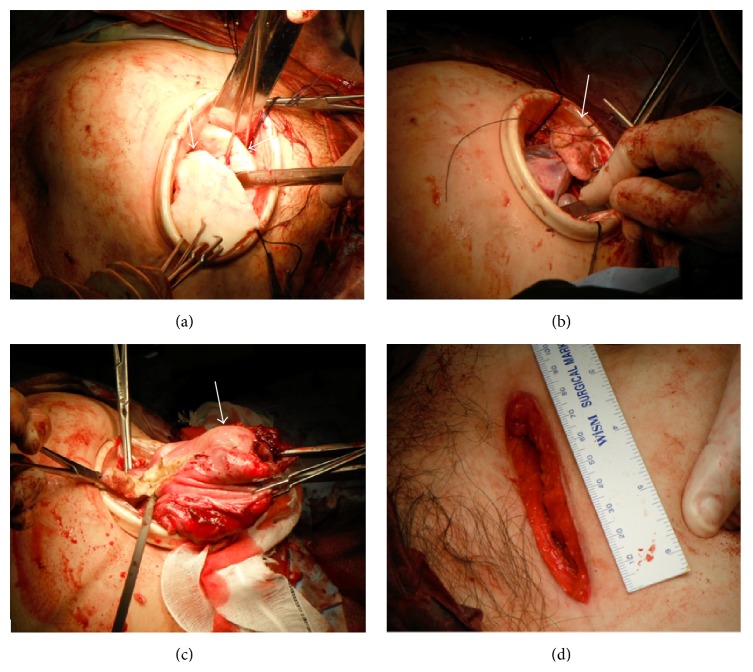
Photographs during the operation. (a) Wedge morcellation of intramural myoma nodule of uterine corpus. (b) Resected peduncle of a subserosal myoma nodule (arrow). (c) Hysterectomy (arrow). (d) The length of the abdominal wound was 8.5 cm at the end of the surgery.

**Figure 4:**
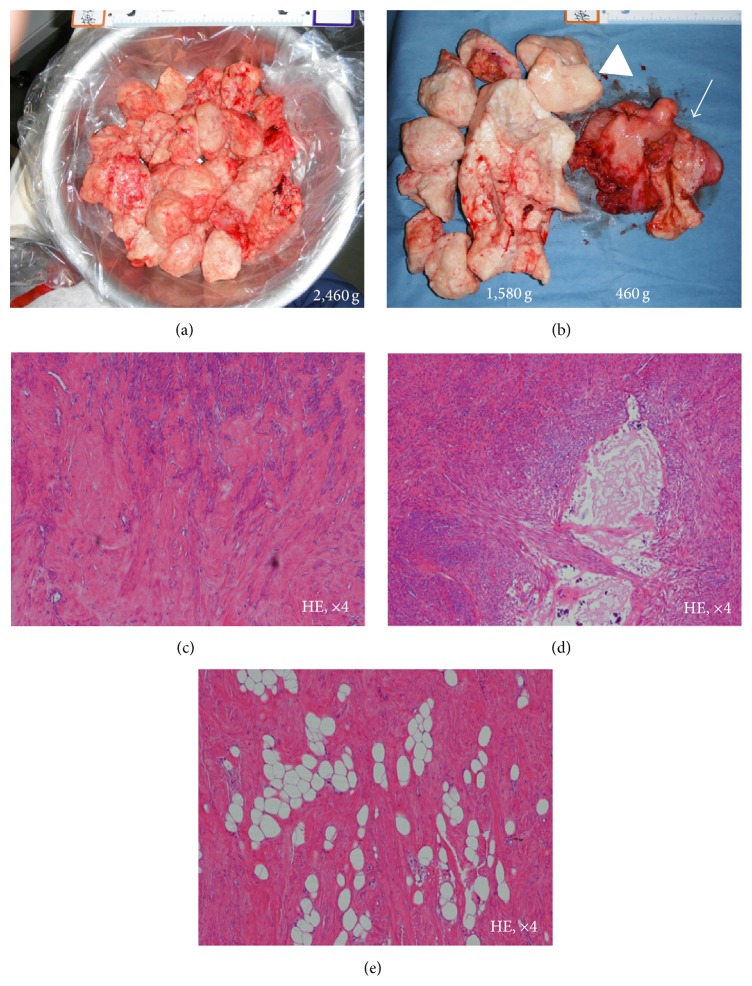
Macroscopic and microscopic finding of resected uterus. The weight of intramural myoma nodule resected from the corpus was 2,460 g (a), and the nodule resected from the uterus was 460 g ((b) arrow), and that resected from the subserosal myoma nodule was 1,580 g ((b) arrow head); thus, the total weight of the resected myoma uteri was 4,500 g. Microscopic findings of myoma nodule show hyaline degeneration (c), mucinous degeneration (d), and lipoleiomyoma (e).

A 44-year-old woman (gravida 0, virgin) consulted our hospital in May 2013 because of the chief complaints of abnormal genital bleeding and hypermenorrhea. Her past history and family history were with no particular event. The patient never had sexual intercourse and thus refused to undergo pelvic examination, rectal examination, and transvaginal ultrasonography. Transabdominal ultrasonography revealed that abdominal solid tumor reached over the navel, hard and movable (Figure 1). We considered myoma uteri because of a MR image (Figure 2) and our data [2]. This method was based on diffusion-weighted magnetic resonance imaging findings and apparent diffusion coefficient values. This diagnostic method can exclude the possibility of leiomyosarcoma if classified in the low-risk group. Her height was 156 cm, weight was 63 kg (body mass index 25.9), anemia (Hb 7.5 g/dL) was revealed, and other blood examination data and electrocardiogram, X-ray of the chest were with no abnormal findings. Her tumor was indication of surgery; to do minilaparotomy hysterectomy with laparoscope was decided because her informed consent was obtained. In June 2013, she was admitted in our hospital and on the same day surgery was done. A 6 cm transverse incision (Maylard incision) was made in the skin above the pubic hairline and was fitted with a self-retaining wound retractor. We tried to observe the abdominal cavity by 10 mm laparoscope through the abdominal wound but cannot see the abdominal cavity because of disturbance of large myoma uteri. The left adnexa can be observed through the abdominal wound; it was cut by Harmonic scalpel and ligated. The right adnexa cannot be observed. In the next step, we performed myomectomy of 2,460 g intramural myoma nodule by morcellation after local injection of vasopressin to uterine wall and made an incision in uterine wall by electric knife, after myomectomy of 2,460 g intramural myoma nodule (Figure 3(a), arrow) (Figure 4(a)), cutting of peduncle of large subserous myoma nodule (Figure 3(b), arrow). After adhesiolysis of right adnexa manually, it was cut by Harmonic scalpel and ligated. After hysterectomy was done (Figure 3(c), arrow) (460 g, Figure 4(b), arrow), morcellation of subserous myoma nodule was done (1580 g, Figure 4(b), arrow head). After checking of hemostasis and gauze count, abdominal wall was closed. The surgery had been finished after confirming the X-ray of the abdominal findings. At the end of surgery, the length of abdominal wound was 8.5 cm (Figure 3(d)), operating time was 128 min, weight of resected myoma uteri was 4,500 g (Figures 4(a) and 4(b)), and intraoperative blood loss was 895 mL. Blood transfusion was not done, and postsurgical course was not problem without anemia. On 1st postoperative day, Hb was 7.8 g/dL (preoperative Hb 9.9 g/dL), and on 4th day, Hb was 8.3 g/dL. The postoperative pain was weak, no additional painkiller without epidural anesthesia. The result of histopathological examination of the excised material was leiomyoma with partial hyalinization, mucinous degeneration, and lipoleiomyoma (Figures 4(c)–4(e)). It seemed that the discharge was possible on 4th postoperative day, but she was discharged on 8th postoperative day because of her hope.

## 3. Discussion

The route of hysterectomy is determined by several factors, for example, weight of uterus, width of vagina, possibility of adhesion, movability of uterus, obesity, existence or not of ovarian tumor, and hope of patient. A weight of uterus is a main factor of preoperative assessment of hysterectomy, and several formulas of calculation of uterine weight are reported. To our knowledge, heaviest uterine weight of each surgical procedure is 2,421 g in vaginal hysterectomy [3], 3,200 g in laparoscopic hysterectomy [3], 3,543 g in robotic hysterectomy [4], and 60,700 g in conventional abdominal hysterectomy (death by pneumonia on 2nd postoperative day) [5]. Therefore, in the present case it was difficult to perform vaginal hysterectomy or laparoscopic hysterectomy; we selected minilaparotomy hysterectomy. To our knowledge, the weight of heaviest uterus was 3,250 g [6] in minilaparotomy hysterectomy and was 3,560 g [7] in minilaparotomy-assisted laparoscopically assisted vaginal hysterectomy. To our knowledge, the present case was heaviest uterine weight that performed minilaparotomy hysterectomy; in other words it seems that 4,500 g of uterine weight is indicated for minilaparotomy hysterectomy (Table 1). A length of abdominal wound of minilaparotomy is reported to be less than 6–10 cm [8–13]. Another author reported that cruciate incision (Kustner incision) is the essence of minilaparotomy hysterectomy [6]. The incision site is usually 1-2 cm below pubic hairline and 2–4 cm above pubic bone in cosmetic sight [10], but a midline incision is not excluded [9, 13]. The length of abdominal incision is often larger than initial incision at start of surgery because a stretch of skin occurred by operative procedures. Thus, it is reported [14] that evaluation of the length of the skin incision should be assessed at the end of operation. In the present case, the length of abdominal incision was 6 cm at the start of surgery, but at the end of surgery the length was extended to 8.5 cm. In other words, 8-9 cm length of incision in minilaparotomy hysterectomy of 4,500 g weighted uterus is necessary. Glasser [6] reported a case of 3,250 g weighted myoma uteri with performed minilaparotomy hysterectomy through 8 cm abdominal incision. Pelosi II and Pelosi III [9] reported that standard minilaparotomy was performed through 3–6 cm length of abdominal incision, and large minilaparotomy was performed through 7-8 cm length. We propose that the length of skin incision in minilaparotomy hysterectomy is <9 cm (usually 6–8 cm) particularly in large uterus. We considered that minilaparotomy hysterectomy is not alternative of vaginal hysterectomy, laparoscopic hysterectomy, or robotic hysterectomy but indicate several large uterine cases which are performed conventional abdominal hysterectomy. There is a limit to minilaparotomy hysterectomy, but we believe that it is possible to perform minilaparotomy hysterectomy up to around 5 kg weighted uterus on the basis of experience of the present case. In this case, the amount of bleeding was greater than the average amount, and the operation time was longer than the average duration. However, we doubted the possibility of performing total laparoscopic hysterectomy or robotic hysterectomy in this case. Based on available literature, we concluded that in this case total laparoscopic hysterectomy and robotic hysterectomy were difficult. If traditional abdominal hysterectomy was performed, a long vertical incision over the navel to inflict an abdominal wound would have been necessary. Therefore, minilaparotomy hysterectomy was beneficial in this case.

In April 2014, US Food and Drug Administration recommended withdrawal of laparoscopic power morcellation [15]. We propose that a case where it is difficult to perform vaginal hysterectomy or laparoscopic hysterectomy and a case where it is necessary to use laparoscopic power morcellation should be considered in order to select minilaparotomy hysterectomy up to around 5 kg weighted uterus for another choice of minimum invasive surgery.

## Figures and Tables

**Table 1:** Heaviest uterine weight removed by various operative procedures.

Operative procedure	Author	Reporting year	Uterine weight (g)	Operating time (min)	Blood loss (mL)	Complication
TAH	Behrend [5]	1930	60,700	—	—	Death of pneumonia 48 h after surgery
TVH	Demir and Marchand [3]	2010	2,421	—	—	—
TLH, LH, LAVH	Demir and Marchand [3]	2010	3,200 (TLH)	360	—	None
Robotically assisted hysterectomy	Silasi et al. [4]	2013	3,543	365	700	—
Minilaparotomy hysterectomy	Glasser [6]	2005	3,250	—	—	—
	Our case	2015	4,500	128	895	None
Minilaparotomy-assisted LAVH	Koh et al. [7]	2008	3,560	150	800	Transfusion (4 units)

TAH, total abdominal hysterectomy; TVH, total vaginal hysterectomy; TLH, total laparoscopic hysterectomy; LAVH, laparoscopically assisted vaginal hysterectomy; LH, laparoscopic hysterectomy.
